# Do Asian breast cancer patients have poorer survival than their western counterparts? A comparison between Singapore and Stockholm

**DOI:** 10.1186/bcr2219

**Published:** 2009-01-24

**Authors:** Benita Kiat Tee Tan, Gek Hsiang Lim, Kamila Czene, Per Hall, Kee Seng Chia

**Affiliations:** 1 Department of General Surgery, Singapore General Hospital, Outram Road, 169608 Singapore, Singapore; 2 Centre for Molecular Epidemiology, c/o Department of Community, Occupational and Family Medicine, Yong Loo Lin School of Medicine, National University of Singapore MD3, 16 Medical Drive, 117597 Singapore, Singapore; 3 Department of Medical Epidemiology and Biostatistics, Karolinska Institutet, P.O. Box 281, SE-171 77 Stockholm, Sweden

## Abstract

**Introduction:**

The difference in breast cancer incidence and prognosis between ethnic groups seeks an explanation. We have recently shown that Swedish women are two to three times more likely to be diagnosed with breast cancer compared with Singaporean women. In the present paper, we compare breast cancer survival in the two countries.

**Methods:**

We compared the survival of 10,287 Singaporean women and 17,090 Swedish women with breast cancer. Relative survival ratios were used to describe the prognosis in the two populations. A Poisson regression model was used to calculate relative risks for different follow-up periods, age groups, time of diagnosis and disease stages.

**Results:**

The majority of the Swedish women had local cancer (80%) compared with Singaporean women (51%). The overall 5-year relative survival of the Swedish women appeared better (80%) than that of the Singaporean women (70%). A similar survival pattern was observed, however, between the two countries in a stage-by-stage comparison. Survival improved for all women in Singapore over the two decades, but only in the premenopausal women in Stockholm. In 1980 to 1989, premenopausal Singaporean women had 27% increased risk of death compared with Swedish women, adjusted for stage and year of follow-up, while the postmenopausal women had 48% increased risk. In 1990 to 1999, this risk decreased by 19% and 22% for the premenopausal and postmenopausal Singaporean women compared with the Swedish women.

**Conclusions:**

The stage-dependent prognosis was similar for Singaporean women and for Swedish women. Singaporean women, both premenopausal and postmenopausal, had pronounced improvement in prognosis over the calendar periods, probably contributed by marked economic improvement, leading to better medical facilities and management with increased awareness of patients to diagnosis and treatment, as well as improved treatment options. Improvement seen only in the premenopausal women in Stockholm was probably due to improved treatment options.

## Introduction

The study of ethnic differences in breast cancer survival is limited except for those between Afro-American women, Asian-American women and Caucasian American women. In the United States, Afro-American women are known to have a worse outcome compared with Caucasian American women. This has been attributed to increased diagnosis of late-stage breast cancers, which could be explained by delayed diagnosis reflecting the socioeconomic status, cultural beliefs, access to healthcare [1], and the proportion of estrogen receptor (ER)-negative tumors in the Afro-American women [2]. In US women diagnosed with breast cancer from 1992 to 1998, a positive ER was found in 81% of non-Hispanic Whites and in 66% of Afro-Americans [3]. ER-negative breast cancer, however, seems to be influenced by parity and age at first birth. It has been reported that increased parity at an early age is associated with ER-negative breast cancer [4]. Early age at first birth could also have an adverse effect on the prognosis of breast cancer [5] due to the interplay of hormonal factors in tumor biology and prognosis. More Afro-American women have children early (<20 years old) [6,7] and tend to have higher parity compared with the non-Hispanic White women [8].

The ethnic disparity in incidence, mortality and survival is evident in the United States, where breast cancer is predominantly a postmenopausal disease in the Caucasian American population [2,9] but a premenopausal disease in Asian or non-Caucasian populations [10-12]. We have recently shown that Swedish women are two or three times more likely to be diagnosed with postmenopausal breast cancer compared with Singaporean women [13]. The difference was most pronounced for older birth cohorts, where it is likely that Singaporean women protected themselves from postmenopausal breast cancer by younger age at first birth and giving birth to many children. The difference in childbearing practices may be of importance for breast cancer prognosis.

Sweden is relatively homogeneous in ethnic stock, while the Singaporean population is multiracial with a prominent Chinese population (76%), 13% Malays and 7% Indians [14]. Over the past four to five decades, Sweden has enjoyed high socioeconomic conditions while Singapore has undergone significant economic restructuring in the 1980s and 1990s. Stockholm and Singapore in recent times have become very similar, being modern city-states with high socioeconomic status, education and living standards and good accessible healthcare systems [15,16]. Sweden has a nationwide mammographic screening program, established since the 1980s. In Singapore, screening was opportunistic until the Singapore Breast Screening program was launched in 2002. Screening mammography has again been proven in more recent studies to decrease mortality in breast cancer [17-20].

These similarities and differences make the study of breast cancer prognosis important. Such study may highlight biological and nonbiological reasons for any difference in the prognosis between Singapore and Sweden. In this paper, we aim to compare the survival in women diagnosed with breast cancer in Singapore and in Sweden in relation to important clinical prognostic markers for breast cancer. We also studied how the trends in survival patterns of the breast cancer patients relate to the change in incidence and mortality.

## Materials and methods

### Study population

All cases of invasive breast cancer diagnosed from 1 January 1980 to 31 December 1999 were obtained from the Singapore and Stockholm cancer registries. Patients with a previous malignancy, including contralateral breast cancer, and those diagnosed with breast cancer at autopsy (death certificate only) were excluded from the study. Follow-up was performed until 31 December 2005 by matching with the national death register. The cause of death was coded in accordance with the International Classification of Diseases and Causes of Death ICD9. The ethical committees at the National University of Singapore and Karolinska Institutet accepted the study without any restrictions. This is normal when de-identified material is used.

### Singapore Cancer Registry

The Singapore Cancer Registry is a population-based registry that was started in 1968. It receives voluntary notifications of incident cancers from all medical practitioners and pathology laboratories, as well as reviews, death certificates and hospital discharges for all patients. Staff of the Registry also reviewed cancer patient hospital discharges and death certificates. The completeness of reporting is high: 96% in the 1970s and close to 100% in the 1990s. The proportion of death-certificate-only notifications was 4.2% for the period 1968 to 1977, 1.0% for 1993 to 1997 and 0.9% for 1998 to 2002 [21]. In total, 4 million individuals inhabit Singapore [14] – 10,287 women diagnosed with breast cancer between 1980 and 1999 were identified and included in the study [21].

### Stockholm Breast Cancer Registry

The Stockholm Breast Cancer Registry was started in 1977 and receives notification of newly diagnosed cases of breast cancer at all departments of oncology and surgery in Stockholm County, which is inhabited by 1.7 million individuals. There were 17,090 women diagnosed with breast cancer recorded in the calendar period 1980 to 1999. None of them were death-certificate-only diagnoses.

### Stage information

The stage of the breast cancer in the Singapore Cancer Registry was classified as localized cancer, regional spread and distant metastases based on the notification forms before 2001. Cancers are staged as local if they are confined entirely to the breast. Regional cancers are those that have extended beyond the limits of the breast directly into surrounding tissues or organs, or into lymph nodes in the region. Distant cancers are those that have spread beyond these locations. No attempt was made to access the extent of localized invasion or the number of regional lymph nodes involved.

Stage information in the Stockholm registry was available according to the TMN staging system: tumor stage, lymph node stage and metastatic spread. This information was reclassified, using the Surveillance, Epidemiology and End Results Comparative Staging Guide for Cancer [22], to be comparable with the Singapore cohort – as localized disease where breast cancer was only identified in the mammary gland; regional disease when there is direct extension to surrounding tissues or organs, or axillary lymph nodes are affected; and distant metastasis.

### Statistical analysis

Age at diagnosis was categorized into six age groups (<35 years, 35 to 44 years, 45 to 54 years, 55 to 64 years, 65 to 74 years, 75+ years) and the period of diagnosis divided into two 10-year periods (1980 to 1989 and 1990 to 1999) to identify change over time; 1990 was selected because the early 1990s were when the use of tamoxifen became widely accepted and adjuvant treatment was more standardized in Singapore.

Descriptive prognostic comparisons between Singaporean women and Swedish women were performed by relative survival analyses. Relative survival ratios were computed by taking the ratio of observed survival to expected survival, accounting for the competing causes of death. The expected survival probabilities were calculated using Ederer II method [23] derived from the general female population from Singapore and Stockholm, respectively, similar to the breast cancer patients in terms of attained age and calendar period of diagnosis. In order to compare the survival between the two countries, cumulative relative survival ratios were age-standardized to the world standard cancer population [24]. A 3-year central moving average for the 5-year relative survival ratios at each calendar year of diagnosis was used to depict the trend across the calendar periods. A Poisson regression model was also used to calculate the excess hazards of death, taking into account the age, disease stage, period of diagnosis, country and years of follow-up. Interactions between country and the age of diagnosis, and between the calendar period and the age of diagnosis, were also analyzed. Two age groups (≤50 years old and >50 years old) were used to represent the premenopausal and postmenopausal age groups in this analysis.

The incidence rates were calculated using the number of invasive breast cancer cases out of the total female population of each country for each time period. The cause of death information was used only to calculate the cause-specific mortality rate, which is the total of breast cancer deaths divided by the total female population of each country. Five-year incidence and mortality rates were reported and were age-standardized to the world standard population [24]. STATA8.2 (StataCorp. College Station, TX: Stata Corporation) was used for the statistical analyses.

## Results

Table 1 presents the characteristics of the women with breast cancer. At the end of 2005, 9,330 (55%) of the Swedish women and 4,782 (46%) of the Singaporean women had died. The median age at diagnosis was stable within each country over the years of diagnosis; however, the Singaporean women were more than a decade younger than those in Stockholm. Stage information was only available for two-thirds of the Singapore cohort. The Swedish women were followed up for a median of 8.7 years (range 0.003 to 26.0 years) while the Singaporean women were followed up for 7.7 years (range 0.003 to 26.9 years)

**Table 1:** Characteristics of the Stockholm and Singapore breast cancer cohorts

Characteristic	Stockholm Breast Cancer Registry	Singapore Cancer Registry
Period of diagnosis	1980 to 1999	1980 to 1999
Number of breast cancer cases	17,090	10,287
1980 to 1989 (%)	7,932 (46)	3,135 (30)
1990 to 1999 (%)	9,148 (53)	7,152 (70)
Median age at diagnosis (years)	62	50
Standard deviation	14	13
Range (years)	18 to 101	12 to 98
Diagnosed ≤ 50 years old (%)	4,247 (25)	5,360 (52)
Diagnosed >50 years (%)	12,843 (75)	4,927 (48)
Number of women with information on disease stage^a ^(%)	16,869 (99)	6,569 (64)
1980 to 1989		
Local cancer (%)	6,047 (77)	1,090 (48)
Regional cancer (%)	1,667 (21)	961 (43)
Distant cancer (%)	165 (2)	196 (9)
1990 to 1999		
Local cancer (%)	7,387 (82)	2,283 (53)
Regional cancer (%)	1,358 (15)	1,692 (39)
Distant cancer (%)	245 (3)	347 (8)
Estrogen receptor status		
Positive	9,874 (77)	Not available
Negative	2,984 (33)	Not available
Number of deaths (%)	9,330 (55)	4,782 (46)
Number of breast cancer deaths (%)	4,050 (24)	2,684 (26)

^a^Invasive cancers are local stage if they are confined entirely to the breast. Regional cancers are those that have extended beyond the limits of the breast directly into surrounding tissues or organs, or into lymph nodes in the region. Distant cancers are those that have spread beyond these localizations.

### Overall survival

**Figure 1:**
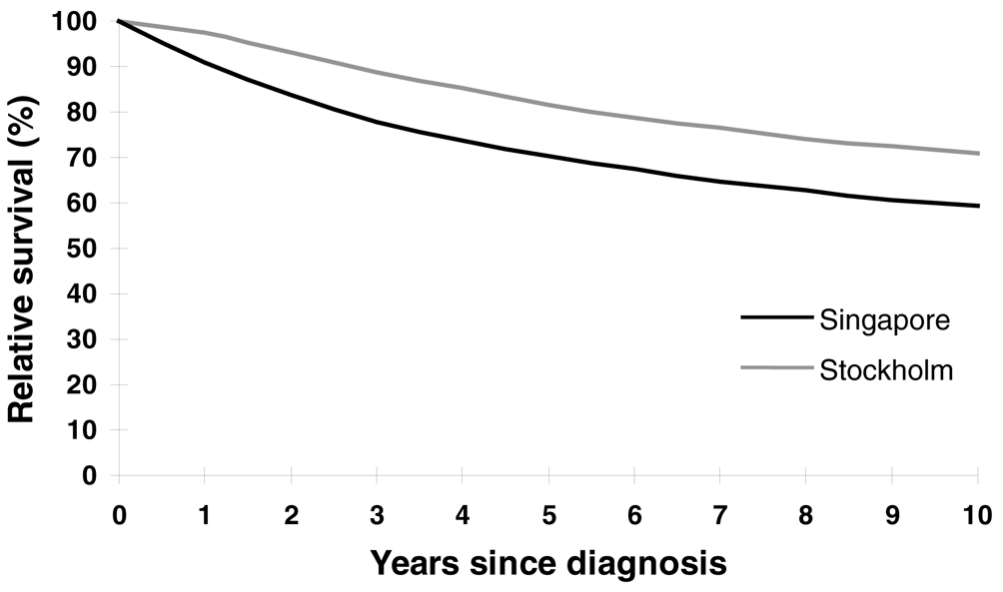
Overall age-standardized relative survival in relation to time since diagnosis. Overall age-standardized relative survival of women diagnosed with breast cancer from 1980 to 1999 in Singapore and in Stockholm in relation to time since diagnosis.

The overall relative survival for women diagnosed with breast cancer from 1980 to 1999 appeared better for the Swedish cohort, where the Swedish women constantly outperformed the Singaporean women at each year of follow-up (Figure 1). The overall age-standardized 5-year relative survival for Singaporean women and for Swedish women was significantly different at 70% and 80%, respectively (Table 2).

**Table 2:** Overall and 5-year age standardized relative survival of women with breast cancer in the Stockholm and Singapore cohorts

Characteristic	Stockholm Breast Cancer Registry		Singapore Cancer Registry	
	
	%	95% confidence interval	%	95% confidence interval
5-year survival rates				
Overall observed survival	72	71 to 74	64	62 to 66
Overall relative survival	80	78 to 82	70	67 to 73
Specific 5-year relative survival				
Local				
1980 to 1989	88	85 to 91	81	71 to 89
1990 to 1999	88	85 to 90	90	85 to 95
Regional				
1980 to 1989	58	51 to 65	47	37 to 57
1990 to 1999	64	57 to 71	68	61 to 75
Distant				
1980 to 1989	19	6 to 37	21	8 to 39
1990 to 1999	21	9 to 37	28	17 to 42

### Survival by stage

**Figure 2:**
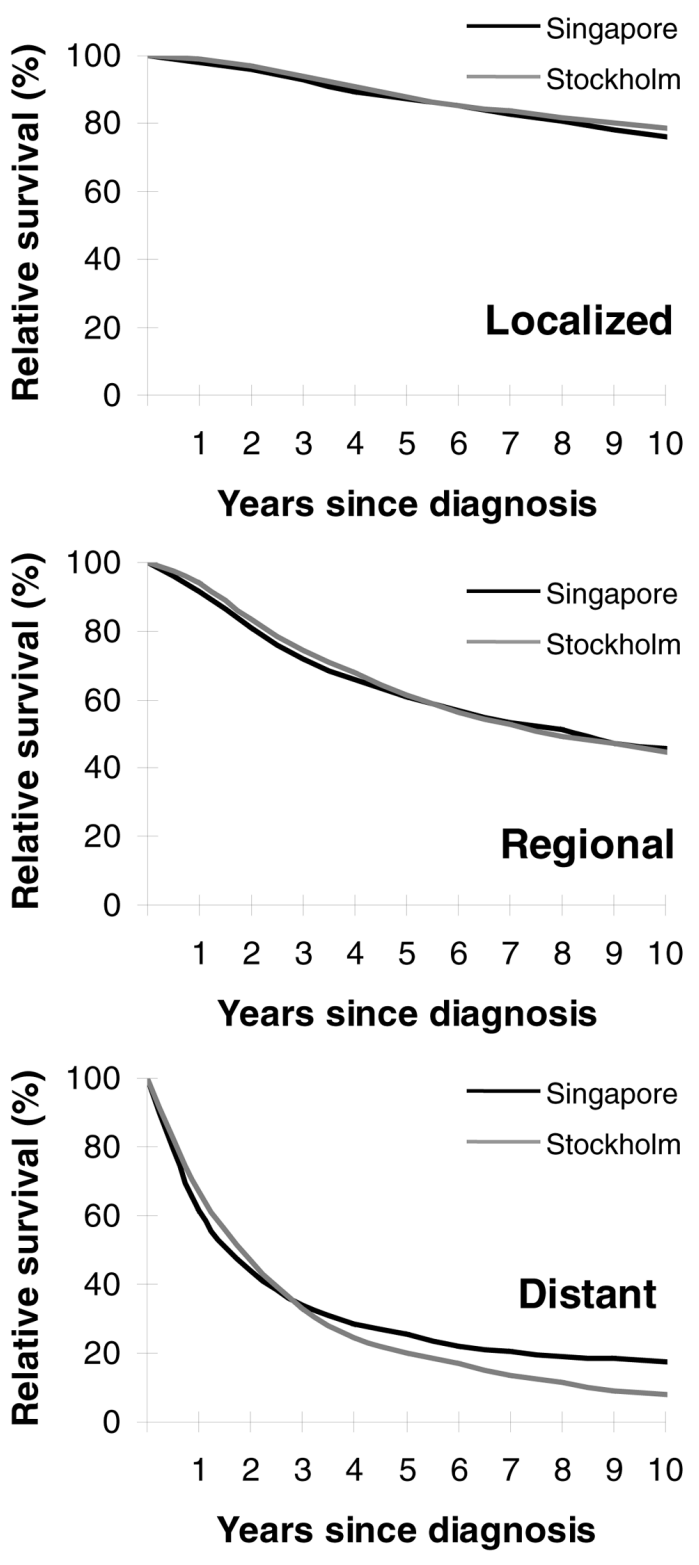
Age-standardized relative survival by the stage of cancer in relation to time since diagnosis. Age-standardized relative survival of women diagnosed with breast cancer from 1980 to 1999 in Singapore and in Stockholm by the stage of cancer in relation to time since diagnosis.

The majority of Swedish patients were diagnosed with a localized cancer while only about one-half of the Singaporean women had localized cancer (Table 1). Over the past 20 years there has been a small increase in women presenting with localized disease with a larger corresponding decrease in regional disease in the Singaporean women; the metastatic cases remained fairly constant (Table 1). When all of the women diagnosed in 1980 to 1999 were stratified by the stage of breast cancer, there was no difference in the relative survival between the two cohorts (Figure 2).

### Survival by period of diagnosis

**Figure 3:**
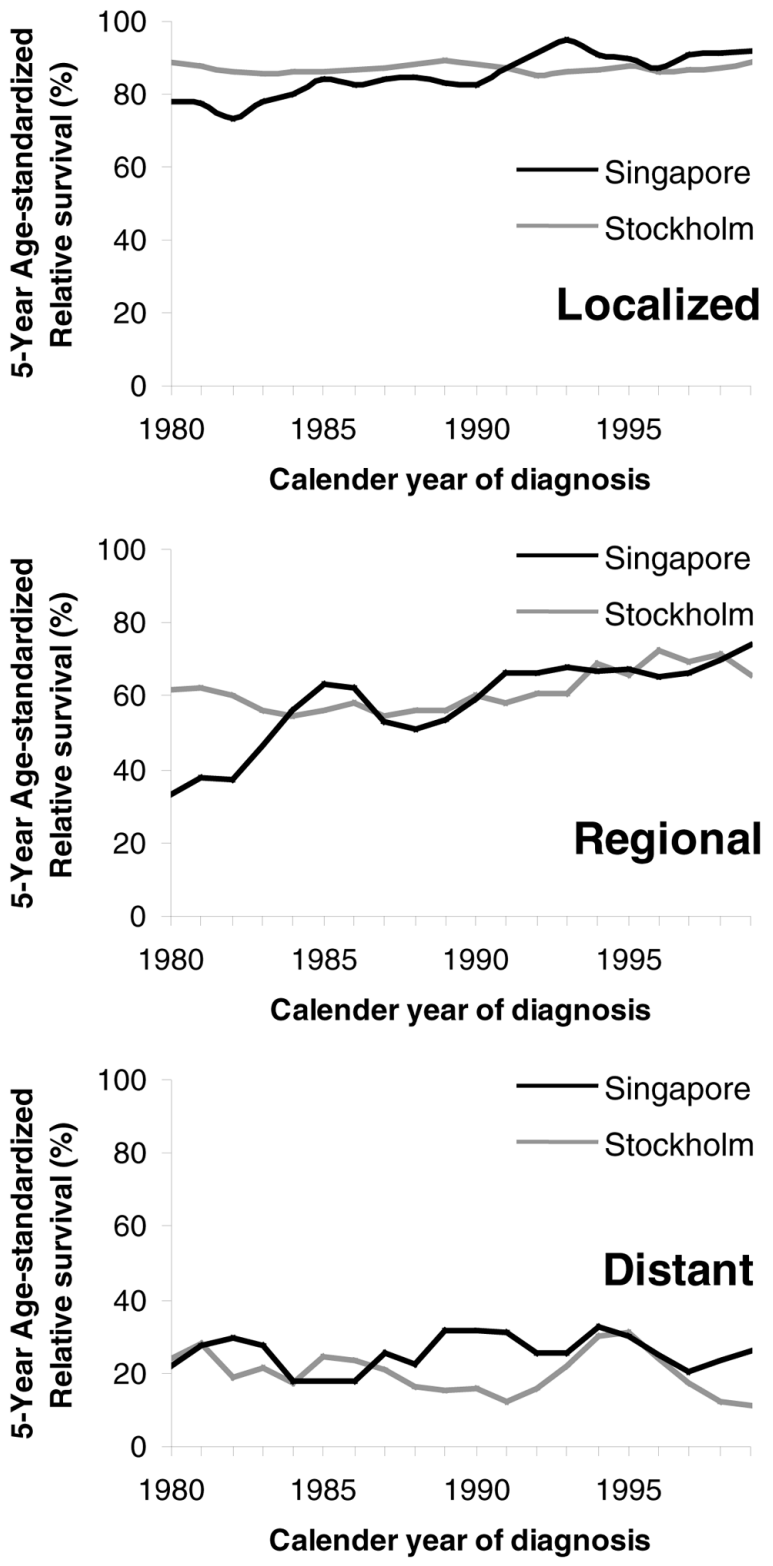
Five-year age-standardized relative survival by stage of breast cancer and calendar year of diagnosis. Five-year age-standardized relative survival of women with breast cancer in Singapore and in Stockholm by the stage of breast cancer and the calendar year of diagnosis (3-year centered moving average).

The 5-year age-standardized relative survival ratio was used to compare the survival by the period of diagnosis between the two cohorts. In the 1980s the Singaporean women showed an improvement in survival across all stages, most marked in those with regional disease (with improvement of almost 20%); a smaller improvement was seen in the Swedish women with the same stage. Singaporean women diagnosed with local cancer and regional cancer had poorer survival than the Swedish women (Table 2 and Figure 3).

In the 1990s survival among the Singaporean women with localized cancer and regional cancer improved and was comparable with the Swedish women. In addition, these Singaporean women with local cancer showed a marginal survival advantage over the Swedish cohort in the later period.

### Survival by age group

Singaporean women were diagnosed with breast cancer earlier in life than the Swedish women; they were on average 10 years younger. While 52% of the Singaporean women diagnosed with breast cancer were 50 years old or younger, only 25% of the Swedish women were 50 years old or younger (Table 1). Over the two decades, the median age in the Stockholm women decreased from 64 to 61 years while it remained stable at 50 years old for the Singaporean women. The prognostic outlook was more optimistic for women with breast cancer when they were between 35 and 75 years old. This was consistent in both countries (Table 3).

**Table 3:** Poisson regression: excess risk of death by stratified by country

	Singapore			Stockholm		
	
	Hazard ratio	95% confidence interval	*P *value	Hazard ratio	95% confidence interval	*P *value
Year of follow-up						
1 year	1.00	(Reference)		1.00	(Reference)	
2 years	1.32	1.14 to 1.51	<0.001	1.69	1.47 to 1.94	<0.001
3 years	1.21	1.04 to 1.40	0.013	1.90	1.65 to 2.18	<0.001
4 years	1.12	0.95 to 1.31	0.178	1.71	1.48 to 1.98	<0.001
5 years	0.96	0.81 to 1.14	0.631	1.84	1.59 to 2.14	<0.001
Age group						
<35 years	1.00	(Reference)		1.00	(Reference)	
35 to 54 years	0.65	0.54 to 0.78	<0.001	0.71	0.57 to 0.88	0.002
45 to 54 years	0.66	0.55 to 0.79	<0.001	0.56	0.45 to 0.69	<0.001
55 to 64 years	0.91	0.75 to 1.10	0.314	0.60	0.49 to 0.74	<0.001
65 to 74 years	0.77	0.62 to 0.96	0.022	0.78	0.64 to 0.96	0.019
75+ years	1.06	0.81 to 1.39	0.673	0.89	0.71 to 1.10	0.281
Stage						
Local	1.00	(Reference)		1.00	(Reference)	
Regional	3.66	3.22 to 4.17	<0.001	4.02	3.66 to 4.41	<0.001
Distant	13.29	11.41 to 15.48	<0.001	17.25	15.09 to 19.71	<0.001
Period of diagnosis						
1980 to 1989	1.00	(Reference)		1.00	(Reference)	<0.001
1990 to 1999	0.53	0.48 to 0.58	<0.001	0.91	0.83 to 0.99	0.027

### Poisson regression

Table 3 presents the risk of death of the women taking age, disease stage, period of diagnosis and years of follow-up into account in each country. As expected, the stage of cancer is an important predictor of survival. The risk of death was decreased in the later period for both countries and improvement was greater in the Singaporean women. As there was a significant interaction between the age at diagnosis and the period of diagnosis (*P *= 0.008), comparison between the two populations was performed stratified by the age and period of diagnosis (Table 4).

**Table 4:** Poisson regression: excess risk of death between countries stratified by age and period of diagnosis

	1980 to 1989			1990 to 1999		
	
	Hazard ratio	95% confidence interval	*P *value	Hazard ratio	95% confidence interval	*P *value
Age ≤ 50 years						
Country^a^						
Stockholm	1.00	(Reference)		1.00	(Reference)	
Singapore	1.26	1.08 to 1.47	0.003	0.81	0.70 to 0.94	0.007
Age >50 years						
Country^a^						
Stockholm	1.00	(Reference)		1.00	(Reference)	
Singapore	1.48	1.29 to 1.69	<0.001	0.78	0.68 to 0.89	<0.001

^a^Adjusted for year of follow-up and disease stage.

In 1980 to 1989 premenopausal Singaporean women had 26% increased risk of death compared with the women in Stockholm, adjusted for stage and year of follow-up, while the postmenopausal women had 48% increased risk. In 1990 to 1999 the Singaporean women experienced a decreased risk of death of 19% and 22%, respectively, for the premenopausal women and the postmenopausal women compared with the Swedish women (Table 4).

In Stockholm, survival improved only in the premenopausal women over the two decades (hazard ratio = 0.74, *P *< 0.001). In Singapore, the improvement was for all women over the same period (hazard ratio = 0.51 and hazard ratio = 0.54 for premenopausal women and postmenopausal women, *P *< 0.0001 and *P *< 0.001, respectively; data not shown).

### Survival in comparison with incidence and mortality rates

**Figure 4:**
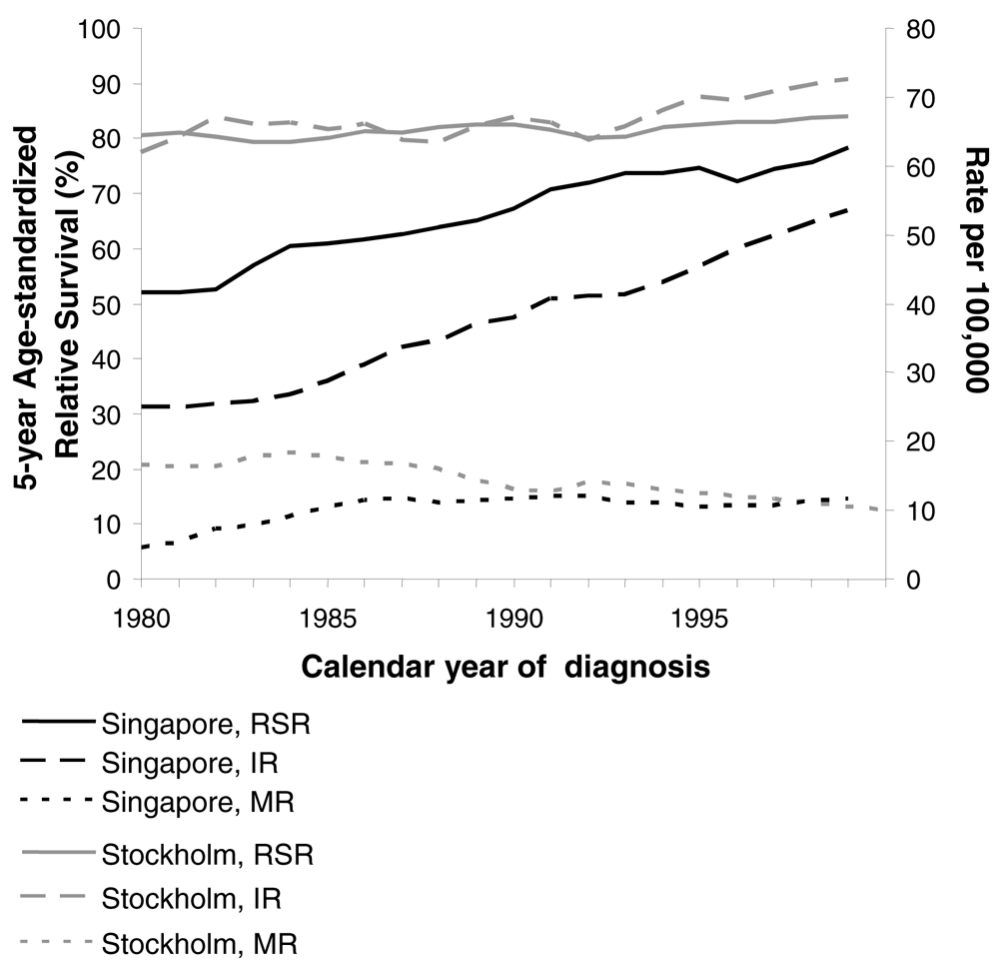
Incidence, 5-year cause-specific mortality and 5-year age-standardized relative survival ratios across the year of diagnosis. Trends in incidence, 5-year cause-specific mortality and 5-year age-standardized relative survival ratios in patients with breast cancer in Singapore and in Stockholm across the calendar year of diagnosis. IR, Incidence Rate; MR, Mortality Rate; RSR, Relative Survival Ratio.

The incidence rate of breast cancer in Singaporean women has more than doubled during the period of study (1980 to 1999). In contrast, the increase in incidence in the Swedish women was only modest. The 5-year cause-specific mortality rate was constant in the Singaporean women from 1985 to 1999 (Figure 4). The mortality rate for the Swedish women also remained relatively constant over the period. The marked improvement in relative survival in the Singaporean women over the two decades is consistent with the discrepancies of the incidence and mortality rates described; the survival in the Swedish women was stable.

## Discussion

Singaporean women diagnosed with breast cancer experienced an overall poorer survival compared with their Swedish counterparts during 1980 to 1999. This poorer survival was confounded by the stage of breast cancer, however, as survival was comparable between the two ethnically different cohorts when stratified by stage. The difference in survival was seen mainly in the regional group in the earlier period, and this converged in the more recent period. Notably, the improvement in survival over the years was marked for the Singaporean women – whereas survival was relatively stable in Stockholm in the postmenopausal women, with improvements only in the premenopausal women.

Confounding factors such as registry completeness, stage migration and distribution, diagnostic improvements and lead-time bias have to be considered when analyzing trends in cancer survival. The strengths of our study include the large number of cases, from population-based registries that report high levels of reliability [21,25]. Women in both countries have individual unique national registration numbers that allow for accurate personal data collection, where women with bilateral breast cancers or multiple cancers can be excluded from the study. The study also extends over two decades, which was probably long enough to observe differences and allow the study of trends.

Incomplete disease stage information for one-third of women in Singapore is a limitation of the present paper. To our knowledge, every effort has been put in to ensure the completeness of cancer reporting over the years. Clinical staging information, which used to be reported voluntarily, could contribute to the lack of information. There is now a follow-up mechanism by the registry, however, to obtain detailed clinical information from the clinical case notes. The proportion of unknown disease stage was hence worst in the early 1990s, only 52% with complete clinical staging for cases in 1990 to 1994 (*P *< 0.001), and this improved in the last 5 years of the study. This incomplete staging is probably random, however, as the age-standardized survival for the Singaporean women with unknown disease stage was comparable with the overall survival of those with stage information (data not shown). The ethnic distribution between the two groups was not different (*P *= 0.576), and the age distribution by the period of diagnosis was also similar except for fewer women between 35 and 54 years old being diagnosed in 1985 to 1989 (*P *= 0.032).

The accuracy of staging could have affected the stage distribution and should be regarded with caution. It is possible that, in the earlier years, node-positive tumors were underdiagnosed and falsely classified as being localized, and hence appeared to have poorer survival. The axillary dissection and histologic assessment of specimens may have been less thorough. The proportion of such cases is unknown in this study, but is probably small. Active screening for distant metastases at the time of initial diagnosis, a practice routinely adopted in Singapore but not in Sweden, can induce stage migration and increase the stage-dependent survival in all stages to the benefit of the Singaporean women; Swedish women with clinically occult distant metastasis may have been understaged. Earlier diagnosis by screening can cause lead-time bias and falsely depict better survival, where the time of diagnosis was earlier while the death from breast cancer was not delayed or avoided. Without a nationwide breast screening program in Singapore, the lead-time bias could not be an advantage for Singapore to explain the decreased risk of death for 1990 to 1999. Opportunistic screening is available and widely used in clinics, however, as healthcare and health awareness improved with the socioeconomic status of Singapore in the 1990s. Length-time bias may exist and is a limitation in this comparison since screening is probably more extensive, having been implemented early in Stockholm. We would expect a more favorable outcome in the Swedish population when a greater proportion of slow-growing breast cancer with good prognosis is being diagnosed [26]. This is difficult to quantify without a randomized trial.

The observation of the overall survival advantage in Stockholm (Figure 1) occurs because the proportion of women diagnosed with a local cancer (80%) was larger than that of Singaporean women (51%) (Table 1). This larger diagnosis is a consequence of established organized mammographic screening in Sweden since the late 1980s, which became nationwide in the mid-1990s. This advantage was present throughout the study period as more Swedish women were consistently being diagnosed with localized disease (Table 1). The effect of stage distribution being a key reason for the difference in survival of the Singaporean population compared with the Stockholm population is reminiscent of a study from Stockholm in the period 1961 to 1973 [27], and is again reflected in a more recent comparison of screened and nonscreened Danish and Swedish populations [28].

When compared across the period of diagnosis, there was decreased risk of death in the premenopausal women in both populations and in the postmenopausal women in Singapore, signifying improvements in both countries. In the later period where nationwide screening was still not present in Singapore, the Singaporean women were performing no worse than the Swedish women (Figure 3). Interestingly, after adjusting for potential confounders, there was a mean 19% (CI 6% to 30%) decrease in risk of death in the premenopausal and a mean 22% (CI 11% to 32%) decrease in the postmenopausal Singaporean women as compared with Swedish women during the period 1990 to 1999 (Table 4).

A significant change in Singapore during the study period is that Singapore underwent much economic restructuring in the 1980s and 1990s, resulting in marked economic improvements over these two decades [14]. The increase in Gross Domestic Product was 725% in Singapore and 190% in Sweden from 1980 to 1999 (International Monetary Fund statistics). This resulted in improved living standards, improved education and presumably better awareness of the disease, and in better healthcare including breast screening – albeit opportunistic – in Singapore. Like Sweden, Singapore enjoys a large network of affordable primary healthcare services that refer to government-funded (Sweden) or heavily subsidized (Singapore) hospital and specialist care services. Healthcare indicators such as life expectancy, the infant mortality rate and the hospital to population ratio are comparable [15,16].

This restructuring coincided with a time trend towards less advanced tumors being diagnosed, where a small but definite increase in women with localized disease and a corresponding decrease in women with regional disease over the study period were observed in Singapore (Table 1). This is also supported by the finding of an increase in incidence of ductal carcinoma *in situ *from 0.4% in 1983 to 1989 to 8.1% in 1999 (Singapore Cancer Registry statistics), an indicator of results of increased mammographic screening. In addition, the improvement in survival in each stage across the periods – including for those with distant metastasis, albeit small (Figure 3 and Table 2) – would suggest improvement in treatment options with the use of adjuvant therapy with antiestrogen and chemotherapy that had become more standardized in the 1990s in Singapore. The quality of the healthcare and treatment routines probably did not differ to a larger extent or to the benefit of Swedish women in the later part of the follow-up period. Treatment details are not available for these patients and are a study limitation. Treatment of breast cancer, however, was standardized under institutional practice in both Stockholm and Singapore in the 1990s, and this was comparable (personal communication with P Hall, Stockholm, and CY Wong, Singapore). The survival equivalence after stratification by stage (Figure 2) suggests that both Singaporean women and Swedish women respond similarly to the treatments given.

Antiestrogen treatment has been well established since the 1990s. Seventy-seven percent of the Swedish women had tumors that were positive for the ER. This is in contrast to a more recent report of 55% Singaporean women from a local institution [29], not different from a series from the largest hospital in Singapore (unpublished data). The Swedish women would be expected to perform better in each stage if the receptor status was indeed stage independent. The present study could not demonstrate the postulated survival advantage of ER-positive tumors, or perhaps the effect of this factor is small. Nevertheless, the improvement of survival only in the premenopausal Swedish women across the period is probably the result of increased aggressiveness in treating this group of women, with tamoxifen as well as chemotherapy.

Until the 1970s Singaporean women had a mean fertility rate of approximately five children compared with two children for Swedish women [5,30]. This higher fertility in the earlier Singaporean birth cohort contributed to the low incidence of breast cancer, especially postmenopausal breast cancer. Unfortunately, this may have resulted in a higher proportion of premenopausal breast cancer (Table 1), which is more likely to be ER-negative with poorer survival. Parity may therefore not only affect risk [4,31-33], but also the prognosis of breast cancer. The present study, however, is limited by the absence of information of the tumor receptor status as well as the unavailability of complete parity information in the Singaporean cohort. It remains unsettled whether risk factors of breast cancer that influence the cancer incidence and malignant phenotype will affect the prognosis.

Other factors such as body mass index, diet, and other behavioral, cultural, environmental, or genetic differences are factors that have been known to affect incidence, but the effect on prognosis is less known [34], and is not available in this study. Singapore, however, reported fewer women who smoke – 3.2% (2004 National Health Survey statistics) compared with 29% of Swedish women in 1980, and 18% in 2005 (Sweden statistics). Only about 6% of postmenopausal women in Singapore are on hormonal replacement therapy for menopause [35], compared with 21% of women in Sweden [36].

As advocated by Dickman and Adami, the trends in survival were interpreted in context with the incidence and mortality rates to evaluate the progress against cancer [37]. As compared with the Swedish women who had enjoyed a more stable economy during this period, the dramatic increase in incidence in Singapore was probably the result of aggressive screening practices that came with increased awareness, better education, and improved healthcare facilities as our economy improved, even without a national screening program. The relatively stable mortality and the corresponding marked increase in the survival ratios in the Singaporean women probably represented improved treatment options. The more superior survival ratios in the earlier years of diagnosis in the Swedish women remained relatively stable, while the increase in incidence coincided with a less dramatic change in the survival compared with the Singaporean women (Figure 4).

## Conclusions

The survival of women with breast cancer in each disease stage was comparable between the Singapore and Stockholm cohorts. We hypothesize that better economic status associated with increased awareness of the disease, better access to screening routines or healthcare quality and options, seen in Stockholm and in Singapore only in the later decade, offer the main explanation for the prognostic differences and similarities.

## Abbreviations

ER: estrogen receptor.

## Competing interests

The authors declare that they have no competing interests.

## Authors' contributions

PH and KSC conceived of the study. BKTT and GHL participated in the study design analysis and carried out the statistical analysis. KC, PH and KSC contributed to the epidemiological aspects and participated in the interpretation of data. All authors contributed to the writing of the manuscript, read and approved the final manuscript and gave final approval of the version to be published.
